# Awareness of and intention to use an online sexually transmitted and blood-borne infection testing service among gay and bisexual men in British Columbia, two years after implementation

**DOI:** 10.17269/s41997-020-00323-4

**Published:** 2020-06-16

**Authors:** Joshun Dulai, Travis Salway, Kimberly Thomson, Devon Haag, Nathan Lachowsky, Daniel Grace, Joshua Edward, Troy Grennan, Terry Trussler, Mark Gilbert

**Affiliations:** 1 Department of Sociomedical Sciences, Mailman School of Public Health, Columbia University, 722 West 168th Street, New York, NY 10032 USA; 2 British Columbia Centre for Disease Control, 655 West 12th Avenue, Vancouver, BC V5Z 4R4 Canada; 3 Faculty of Health Sciences, Simon Fraser University, 888 University Drive, Burnaby, BC V5A 1S6 Canada; 4 School of Population and Public Health, University of British Columbia, 2206 East Mall, Vancouver, BC V6T 1Z3 Canada; 5 School of Public Health & Social Policy, University of Victoria, 3800 Finnerty Road, Victoria, BC V8P 5C2 Canada; 6 Social & Behavioural Health Sciences Division, Dalla Lana School of Public Health, University of Toronto, 155 College Street, Toronto, ON M5T 3M7 Canada; 7 Health Initiative for Men, 1033 Davie Street, Vancouver, BC V6E 1M5 Canada; 8 Division of Infectious Diseases, Faculty of Medicine, University of British Columbia, 2194 Health Sciences Mall, Vancouver, BC V6T 1Z3 Canada; 9 Community-Based Research Centre, 808 Nelson Street, Vancouver, BC V6Z 2H2 Canada

**Keywords:** Men who have sex with men, Gay and bisexual men, Sexually transmitted and blood-borne infection testing, Online sexual health services, Hommes ayant des relations sexuelles avec des hommes, Hommes gais et hommes bisexuels, Dépistage des infections transmissibles sexuellement et par le sang, Services de santé sexuelle en ligne

## Abstract

**Objectives:**

This study assessed gay, bisexual, and other men who have sex with men’s (GBMSM) awareness of and intention to use GetCheckedOnline, an online sexually transmitted and blood-borne infection (STBBI) testing service.

**Methods:**

A cross-sectional study was conducted two years after launch among GBMSM > 18 years of age in British Columbia, Canada. Participants were recruited through community venues, clinics, websites, and apps.

**Results:**

Of 1272 participants, 32% were aware of GetCheckedOnline. Gay identity, regularly testing at an STBBI clinic, being out to one’s healthcare provider, attending GBMSM community venues, and frequent social media use were associated with awareness. Among participants who were aware but had not used GetCheckedOnline, knowing GetCheckedOnline users, using social media, not knowing where else to test, and not wanting to see a doctor were associated with intention to use GetCheckedOnline.

**Conclusion:**

Early promotion of GetCheckedOnline resulted in greater awareness among those connected to GBMSM.

## Résumé

**Objectifs:**

Évaluer chez les hommes gais, bisexuels et les hommes ayant des relations sexuelles avec des hommes (gbHARSAH) la connaissance de GetCheckedOnline, un service de dépistage en ligne des infections transmissibles sexuellement et par le sang (ITSS), et l’intention d’utiliser ce service.

**Méthode:**

Deux ans après le lancement du service, une étude transversale a été menée auprès d’hommes gbHARSAH de plus de 18 ans en Colombie-Britannique, au Canada. Les participants ont été recrutés dans les milieux associatifs, les cliniques, sur des sites Web et au moyen d’applications.

**Résultats:**

Sur 1 272 participants, 32 % connaissaient GetCheckedOnline. L’identité gaie, le dépistage périodique à une clinique d’ITSS, le fait d’avoir dévoilé son orientation sexuelle à son dispensateur de soins de santé, la fréquentation de milieux associatifs pour hommes gbHARSAH et l’utilisation fréquente des médias sociaux étaient associés à la connaissance du service. Chez les participants qui connaissaient GetCheckedOnline mais qui ne l’avaient pas utilisé, le fait de connaître des utilisateurs de GetCheckedOnline, l’utilisation des médias sociaux, le fait de ne pas savoir où se faire tester ailleurs et le fait de ne pas vouloir voir un médecin étaient associés à l’intention d’utiliser GetCheckedOnline.

**Conclusion:**

La promotion précoce de GetCheckedOnline a rehaussé la visibilité de ce service dans les milieux en lien avec les hommes gbHARSAH.

## Introduction

Gay, bisexual, and other men who have sex with men (GBMSM) are disproportionately affected by sexually transmitted and blood-borne infections (STBBI; British Columbia Centre for Disease Control 2018a, b) and face distinct barriers to STBBI testing (Gilbert et al. 2013; Hottes et al. 2012). Decreasing barriers to testing may reduce the impact and onward transmission of STBBIs in this population by providing prompt diagnosis and treatment (i.e., secondary prevention). Online services allow individuals to receive STBBI tests without having to visit a clinic and thus are one promising method for reducing barriers and increasing testing among GBMSM. Online STBBI testing services vary in process, although typically clients answer screening questions over the Internet instead of in person with a doctor or nurse. They then either order a self-testing kit online and return their specimens in the mail, or download a laboratory requisition and provide specimens on-site in a non-clinic-based setting. Clients receive their results online (Gibbs et al. 2018; Greenland et al. 2011; Koekenbier et al. 2008; Loos et al. 2016; Lorimer and McDaid 2013; Platteau et al. 2015), over the phone (Chai et al. 2010; Gaydos et al. 2006), or through text message (Woodhall et al. 2012).

In this context, the British Columbia Centre for Disease Control (BCCDC) launched *GetCheckedOnline* (GCO) in September 2014 as an alternative to in-person testing options already in place in the province. The development of GCO has been described in detail elsewhere (Gilbert et al. 2016). In brief, GCO clients first create an online account using a promotional code. To request online STBBI tests, clients answer a series of sexual history questions to determine the appropriate tests to recommend. Based on these recommendations, clients then confirm the tests they want and print a lab requisition. They then go to a participating laboratory location to collect and submit samples for HIV, syphilis, gonorrhea, chlamydia, and/or hepatitis C testing; clients receive their results within 1 to 2 weeks. If any results are positive, or if there are issues with the sample (e.g., sample container damaged), results are not available online, and a nurse contacts the client directly to arrange treatment or retesting. Test results that are negative for STBBIs are provided online. GCO was initially promoted to GBMSM in the Greater Vancouver area in Canada through a promotion campaign from April to August 2015 with promotion on gay websites and apps and in community venues accessed by GBMSM (Gilbert et al. 2019).

Our team previously surveyed an online sample of Canadian GBMSM in 2011/2012 and found that 72% intended to use a hypothetical Internet-based testing service, if available (Gilbert et al. 2013). While measures of intention for health behaviour are useful in preliminary stages of intervention planning, intention is an imperfect proxy for behaviour (Todd et al. 2016); thus, follow-up studies are needed to determine uptake. More specifically, it is important from an implementation perspective to understand how awareness of the intervention has diffused through a population (in this case, GBMSM in British Columbia (BC)) and what additional factors should be emphasized in future promotion of the service (e.g., among those aware of but unengaged with the service). Moreover, fundamental causes theory predicts that socially disadvantaged groups stand to miss opportunities to use new interventions, owing to lower access to social or material resources, including those related to healthcare interventions (Phelan et al. 2010). In this context, it is additionally important to measure social differences in awareness of and intention to use new interventions, such as online STBBI testing.

Therefore, the primary objectives of this study were to assess awareness of GCO two years after implementation and intention to use GCO among those aware of the service, and to assess whether GCO was addressing or had the potential to address health inequities that exist within subgroups of GBMSM in relation to uptake of online STBBI testing. Our overarching goal was to use this evidence to inform future promotion of GCO and related online STBBI interventions.

## Methods and materials

### Eligibility criteria and recruitment

This report is based on a cross-sectional survey of self-identified GBMSM, 18 years of age or older and living in BC. Data collection began on July 29, 2016, and ended December 18, 2016 (~ 5 months), which was 23–28 months after GCO launched. No personally identifiable information was collected, and participation was voluntary. For this study, venue-based (non-probability) sampling was used to recruit GBMSM. However, to try to obtain as diverse a sample of this community as possible, a multi-pronged sampling strategy was devised to recruit GBMSM through three venue categories: GBMSM community venues, STBBI clinics targeting GBMSM, and GBMSM online/mobile sites/apps. Paper-based surveys were distributed at pride events, festivals, and GBMSM bars (hereafter, “community venues”) in the Greater Vancouver region during the early weeks of data collection. Paper survey data were also collected at local STBBI clinics operated by the Health Initiative for Men (HIM), a community organization that was created to address the health needs of GBMSM living in Metro Vancouver (hereafter, “HIM clinics”). HIM operates five sexual health centres where GBMSM can make appointments or walk in to get STBBI testing, access HIV non-occupational post-exposure prophylaxis (PEP), and receive treatment for STBBIs. Surveys were administered at all five Metro Vancouver locations.

Finally, an online version of the survey was distributed through six different online and mobile app channels: Craigslist; Scruff; Squirt; Hornet; boosts on the HIM Facebook page; as well as social media platforms, websites, and e-mail lists of community organizations located within the province that cater to GBMSM (hereafter, grouped as “online” venues). The online survey was created using the program FluidSurveys.

### Survey development

The survey instrument was developed by the study team at the BCCDC with input from other researchers and community partners. The survey was pilot tested with a small group of volunteers and revised before being used to collect data from participants. Questions were adapted from past or current surveys of GBMSM or about GCO, with new questions created for our specific purpose. The Diffusions of Innovations (DOI) theoretical framework was used in designing the survey and its questions (Rogers 2005). This theory seeks to explain how novel ideas or interventions spread along various modes of communication over time within a population. Berwick suggests that there are three important factors that determine how quickly an innovation is spread: (a) perception of the innovation, (b) characteristics of the people who adopt the innovation or fail to do so, and (c) contextual factors (Berwick 2003). Accordingly, the survey included the following three outcomes: awareness, use, and intention to use GCO. In addition, we measured the following domains corresponding to DOI predictor variables: perceived benefits and drawbacks of GCO (DOI category: *perception of the innovation*); usual STBBI testing location and satisfaction with said location, specific barriers to STBBI testing, and knowledge of GCO users (DOI category: *contextual factors*); and STBBI testing history; sexual orientation disclosure to healthcare providers; number of sexual partners within the last year; technology use; attendance at specific GBMSM community venues in the past year; and demographic information (DOI category: *characteristics of the adopters*).

### Outcomes of interest

Survey items were selected to measure awareness of and intention to use GCO among GBMSM living in BC. Awareness was assessed by providing a brief description of GCO and asking the question: “Before today did you know about GetCheckedOnline?” Intention to use GCO was assessed with the question: “How unlikely or likely is it that you will get tested through *GetCheckedOnline* in the future?” Five response options were offered: “Very likely”, “Likely”, “Neither likely nor unlikely”, “Unlikely”, and “Very unlikely”. For both univariate and multivariable analyses, this item was recoded as the binary variable “intention to use GCO” with those who reported being “Very Likely” or “Likely” categorized as those who “intend to use” GCO and all other responses were recoded as those who “do not intend to use” GCO.

### Analysis plan

**Fig. 1:**
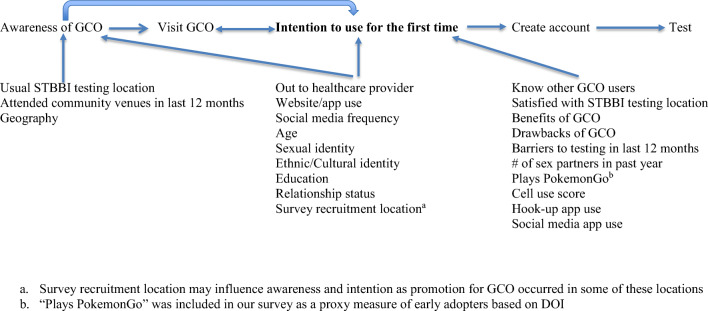
A conceptual causal diagram of the hypothesized relationships for variables of interest. ^a^Survey recruitment location may influence awareness and intention as promotion for GCO occurred in some of these locations. ^b^“Plays PokemonGo” was included in our survey as a proxy measure of early adopters based on DOI

To identify potential explanatory variables associated with awareness of and intention to use GCO among those aware of the service, a conceptual causal diagram was created, informed by DOI theory (see Fig. 1; Berwick 2003; Rogers 2005). A continuous score representing mobile app and website use termed “social media app score” was created by counting the number of apps and websites used by the participant. Two sets of binary logistic regression analyses were conducted. First, we evaluated associations between explanatory variables and awareness of GCO in the full sample. Second, we evaluated associations between explanatory variables and intention to use GCO, restricted to those who were aware of GCO but had not yet used it. These analyses were applied in the interest of targeting future GCO promotion including strategies for reaching GBMSM unaware of GCO, and identifying messages that may increase intention to use among GBMSM who have heard about GCO but have not yet used the service. Univariate odds ratios (uOR) and adjusted odds ratios (aOR) were estimated, and 95% confidence intervals excluding 1 were considered statistically significant. All hypothesized explanatory variables—identified using the causal diagram—were entered into the multivariable model simultaneously to avoid omission of negatively confounded variables that may be missed with forward/stepwise selection procedures (Vittinghoff et al. 2012). In interpreting results, we emphasize uOR rather than aOR because unadjusted measures are more relevant for our goal of informing future promotion of GCO; i.e., adjusted measures may obscure meaningful differences in awareness of and intention to use GCO, due to confounding. All data were analyzed using SPSS Version 21. This study was approved by the Ethics Board at the University of British Columbia.

## Results

In total, 1272 GBMSM participated in this study. The proportion of men recruited from each venue were as follows: 24.1% (307/1272) from community venues, 23.8% (303/1272) from HIM clinics, and 52.0% (662/1272) from online venues. Sample demographic characteristics are presented in Table 1. The majority of study participants were under the age of 40 (53.5%, 646/1272): 26.2% (316/1207) were 18–29, and 27.3% (330/1207) were 30–39. In total, 78.1% (987/1264) identified as gay. Most of the study participants lived within the city of Vancouver (55.3%, 704/1272) with another 24.5% (312/1272) living in the surrounding cities comprising Metro Vancouver, and 12.3% (156/1272) living in other parts of the province. Most study participants identified as White (73.0%, 917/1256), and East Asian (9.0%, 113/1256) and Latino/Hispanic (5.9%, 74/1256) were the most frequently reported racialized minority identities. Indigenous participants comprised 2.9% (37/1256) of the sample. The overall study sample was highly educated, with 67.4% (848/1258) of participants having completed some form of post-secondary education. A majority of participants were single (52.4%, 657/1255).

**Table 1:** Characteristics of GBMSM who participated in a survey about Internet-based STBBI testing in BC, 2016

Variables	*n/N* (%)
Age (birth year)	
18–29 (< 1986)	316/1207 (26.2%)
30–39 (1986–1977)	330/1207 (27.3%)
40–49 (1976–1967)	208/1207 (17.2%)
50–59 (1966–1957)	205/1207 (17.0%)
60+ (1956+)	148/1207 (12.3%)
Sexual identity	
Gay (homosexual)	987/1264 (78.1%)
Bi (bisexual)	202/1264 (16.0%)
Straight (heterosexual)	60/1264 (4.7%)
Queer	59/1264 (4.7%)
Two-spirit	12/1264 (0.9%)
Other	14/1264 (1.1%)
Prefer not to say	13/1264 (1.0%)
Region	
Vancouver	704/1272 (55.3%)
Suburban Vancouver	312/1272 (24.5%)
Rest of BC	156/1272 (12.3%)
Invalid postal code	100/1272 (7.9%)
Ethnic/cultural identity	
Indigenous (First Nations, Inuit, Métis)	37/1256 (2.9%)
African	8/1256 (0.6%)
East Asian	113/1256 (9.0%)
South Asian	31/1256 (2.5%)
Southeast Asian	44/1256 (3.5%)
Caribbean	12/1256 (1.0%)
Latino/Hispanic	74/1256 (5.9%)
Middle Eastern	24/1256 (1.9%)
Pacific Islander	16/1256 (1.3%)
White/Caucasian	917/1256 (73.0%)
Other	33/1256 (2.6%)
Prefer not to say	8/1256 (0.6%)
Education	
Some high school	19/1258 (1.5%)
High school	110/1258 (8.7%)
Some college/university	281/1258 (22.3%)
College	184/1258 (14.6%)
University degree (BA, BSc, etc.)	429/1258 (34.1%)
Graduate degree (MA, MBA, etc.)	192/1258 (15.3%)
Doctorate (PhD, MD, etc.)	43/1258 (3.4%)
Relationship status	
Single	657/1255 (52.4%)
Married to a man	125/1255 (10.0%)
Partnered with a man but not married	298/1255 (23.7%)
Separated, divorced, or widowed from a man	21/1255 (1.7%)
Married to a woman	62/1255 (4.9%)
Partnered with a woman but not married	35//1255 (2.8%)
Separated, divorced, or widowed from a woman	35/1255 (2.8%)
Other	22/1255 (1.8%)
Usual STBBI testing location	
Family physician	439/1267 (34.6%)
Walk-in medical clinic	211/1267 (16.7%)
Public health STBBI clinic	290/1267 (22.9%)
Youth clinic	22/1267 (1.7%)
Health Initiative for Men (HIM) clinic^a^	457/1267 (36.1%)
At a hospital or emergency room	49/1267 (3.9%)
Through GetCheckedOnline	21/1267 (1.7%)
Other	80/1267 (6.3%)
No usual place	51/1267 (4.0%)
I’ve never been tested for STBBIs	63/1267 (5.0%)
Satisfied with current STBBI testing location	1001/1247 (80.3%)
Barriers to STBBI testing in the last 12 months	
None of the above	610/1173 (52.0%)
The wait was too long	191/1173 (16.3%)
Needed an appointment	174/1173 (14.8%)
The clinic wasn’t open when I could test	170/1173 (14.5%)
Other	86/1173 (7.3%)
Couldn’t get anonymous testing	85/1173 (7.2%)
The clinic was too far away	84/1173 (7.2%)
Didn’t want to see a doctor or nurse	76/1173 (6.5%)
Didn’t know where to go	73/1173 (6.2%)
I do not like needles	42/1173 (3.6%)
Out to healthcare provider	
Yes	906/1255 (72.2%)
No	242/1255 (19.0%)
Not sure	34/1255 (2.7%)
Do not have a healthcare provider or have not seen one	73/1255 (5.7%)
Cell phone use	
Texting	1171/1260 (92.9%)
Surfing the Internet	1084/1260 (86.0%)
Using other apps (navigation, games, etc.)	915/1260 (72.6%)
Meeting guys (Grindr, Scruff, etc.)	794/1260 (62.4%)
Playing Pokémon Go	267/1260 (21.2%)
I have a cell phone but do not do any of the above	34/1260 (2.7%)
I do not have a cell phone	30/1272 (2.4%)
Website/app use	
Facebook	865/1223 (70.7%)
YouTube	793/1223 (64.8%)
Grindr	565/1223 (46.2%)
Instagram	544/1223 (44.5%)
Craigslist	461/1223 (37.7%)
Squirt	401/1223 (32.8%)
Scruff	374/1223 (30.6%)
Twitter	341/1223 (27.9%)
Snapchat	321/1223 (26.2%)
Hornet	177/1223 (14.5%)
Manhunt	158/1223 (12.9%)
Growlr	110/1223 (9.0%)
Jack’d	108/1223 (8.8%)
BarebackRT	101/1223 (8.3%)
Frequent social media use^b^	1068/1255 (85.1%)
Attended community venues in the last 12 months (versus not)	
Gay bar, pub, or nightclub	713/1005 (70.9%)
Bathhouse	205/1005 (20.4%)
LGBT organization	374/1005 (37.2%)
Gay social, recreational, or sporting group	293/1005 (29.2%)
Vancouver pride festival or event	741/1005 (73.7%)
Other community events for LGBT individuals	239/1005 (23.8%)
Number of sex partners in the last 12 months	
0	64/1177 (5.4%)
1	184/1177 (15.6%)
2–5	401/1177 (34.1%)
6–9	150/1177 (12.7%)
10+	378/1177 (32.1%)
Survey recruitment location	
Community venues	307/1272 (24.1%)
HIM clinics	303/1272 (23.8%)
Online	662/1272 (52.0%)
Aware of GCO	411/1268 (32.4%)
Unaware of GCO	857/1268 (67.6%)
How heard about GCO^b^	
Ad on a website or phone app	130/391 (33.2%)
Printed material (posters, brochures, etc.)	84/391 (21.5%)
Social media (Facebook, Twitter, etc.)	81/391 (20.7%)
From friends	70/391 (17.9%)
From a physician, nurse, or clinic	59/391 (15.1%)
From someone at a community organization	54/391 (13.8%)
Other	54/391 (13.8%)
News media (TV, newspaper, Xtra, etc.)	43/391 (11.0%)
From a boyfriend/partner	14/391 (3.6%)
Know any other GCO Users^c^	84/391 (21.5%)
Visited GCO website^c^	174/388 (44.8%)
Created an account with GCO^c^	68/388 (17.5%)
Tested through GCO^c^	44/388 (11.3%)
Intend to use GCO	605/1248 (48.5%)
Intention to use GCO among those aware and who have not tested through GCO	166/367 (45.2%)
Benefits of GCO	
Testing without waiting for an appointment	631/1255 (50.3%)
Getting test results online	572/1255 (45.6%)
Saves time	471/1255 (37.5%)
Easier to go to a lab than a clinic	416/1255 (33.1%)
Do not need to see a doctor or nurse	415/1255 (33.1%)
Testing when the clinic is full	372/1255 (29.6%)
Do not need to talk about my sex life	368/1255 (29.3%)
Not sure	243/1255 (19.4%)
Do not need to get a physical exam	223/1255 (17.8%)
I see no benefits	76/1255 (6.1%)
Other	42/1255 (3.3%)
Drawbacks of GCO	
Not speaking with a doctor or nurse	480/1247 (38.5%)
Unclear how it works	324/1247 (26.0%)
Worried about the privacy of my online information	244/1247 (19.6%)
Not sure	217/1247 (17.4%)
I see no drawbacks	177/1247 (14.2%)
Need to print the lab form	172/1247 (13.8%)
Need to have a promo code to sign up	111/1247 (8.9%)
Not easy to get to a lab	99/1247 (7.9%)
Not comfortable going to a lab	86/1247 (6.9%)
Low trust in the service	70/1247 (5.5%)
Other	62/1247 (5.0%)
Getting test results online	59/1247 (4.7%)

^a^Local STBBI clinic used by GBMSM

^b^“Frequent” defined as daily use or using social media a few times a week

^c^Among those aware

Regarding the study outcome measures, 32.4% (411/1268) of participants reported being aware of GCO (see Table 1). Among those aware of GCO, 44.8% (174/388) had visited the website, 17.5% had created an account (68/388), and 11.3% (44/388) had tested; thus, overall, 3.5% of GBMSM surveyed (44/1268) had tested for STBBIs using GCO. Among those aware of the service and who had not yet tested through GCO, 45.2% (166/367) reported intending to do so. Among those who had used the service (*n* = 44), 88.6% reported intending to use the service again.

Factors associated with awareness of GCO are shown in Table 2. Being out to one’s healthcare provider (uOR 2.29 (1.67–3.14)), website/app use (uOR 1.36 (1.07–1.73)), and frequent social media use (uOR 1.84 (1.29–2.62)) were all associated with greater odds, and bisexual identity (uOR 0.67 (0.47–0.96)) with lower odds, of awareness of GCO with CI that excluded uOR of 1. Participants who usually tested at a public health STBBI clinic (uOR 1.57 (1.20–2.06)), youth clinic (uOR 2.54 (1.09–5.93)), or HIM clinic (uOR 2.44 (1.91–3.11)) had higher odds of awareness, while those who usually tested through a family doctor or hospital (uOR 0.39 (0.18–0.85)), and those who had no usual testing location (uOR 0.27 (0.12–0.65)) or history of testing (uOR 0.29 (0.14–0.61)) had lower odds of awareness, with CI that excluded uOR of 1. All community venue variables were also associated with awareness with the exception of the bathhouse variable.

**Table 2:** Characteristics associated with awareness of GetCheckedOnline in a survey of GBMSM in BC, 2016 (*n* = 1272)

Variables	Aware *n/N* (%)	Unaware *n/N* (%)	uOR (95% CI)	aOR (95% CI)
Intend to use GCO	205/605 (33.9%)	400/605 (66.1%)	NA	NA
Usual STBBI testing location^a^				
Family physician	123/436 (28.2%)	313/436 (71.8%)	0.74 (0.57–0.95)	0.75 (0.51–1.10)
Walk-in medical clinic	64/211 (30.3%)	147/211 (69.7%)	0.89 (0.65–1.22)	0.77 (0.49–1.19)
Public health STI clinic	117/290 (40.3%)	173/290 (59.7%)	1.57 (1.20–2.06)	1.57 (1.09–2.27)
Youth clinic	12/22 (54.5%)	10/22 (45.5%)	2.54 (1.09–5.93)	1.81 (0.66–4.98)
Health Initiative for Men (HIM) clinic	206/456 (45.2%)	250/456 (54.8%)	2.44 (1.91–3.11)	1.64 (1.13–2.37)
At a hospital or emergency room	8/49 (16.3%)	41/49 (83.7%)	0.39 (0.18–0.85)	0.28 (0.10–0.84)
Other	31/80 (38.8%)	49/80 (61.3%)	1.34 (0.84–2.14)	1.23 (0.69–2.21)
No usual place	6/50 (12.0%)	44/50 (88.0%)	0.27 (0.12–0.65)	0.40 (0.11–1.43)
I’ve never been tested for HIV/STIs	8/63 (12.7%)	55/63 (87.3%)	0.29 (0.14–0.61)	1.97 (0.60–6.44)
Out to healthcare provider	331/903 (36.7%)	572/903 (63.3%)	2.29 (1.67–3.14)	1.92 (1.18–3.10)
Website/app use	248/700 (35.4%)	452/700 (64.6%)	1.36 (1.07–1.73)	1.29 (0.92–1.80)
Frequent social media use	366/1065 (34.4%)	699/1065 (65.6%)	1.84 (1.29–2.62)	1.87 (1.10–3.17)
Attended community venues in last 12 months (versus not)^a^				
Gay bar, pub, or nightclub	269/710 (37.9%)	441/710 (62.1%)	1.58 (1.17–2.13)	1.19 (0.82–1.72)
Bathhouse	81/204 (39.7%)	123/204 (60.3%)	1.29 (0.94–1.77)	1.18 (0.81–1.72)
LGBT organization (HIM, Qmunity, etc.)	178/373 (47.7%)	195/373 (52.3%)	2.42 (1.85–3.17)	1.59 (1.14–2.21)
Gay social, recreational, or sporting group; Vancouver pride festival or event; other community events for LGBT individuals	339/966 (35.1%)	627/966 (64.9%)	1.73 (1.29–2.32)	0.57 (0.23–1.44)
Age				
Continuous, years	Median: 35 (SD:13.7)	Median: 39 (SD: 14.2)	0.99 (0.98–1.00)	1.00 (0.98–1.01)
18–29 (< 1986)	114/316 (36.1%)	202/316 (63.9%)		
30–39 (1986–1977)	118/329 (35.9%)	211/329 (64.1%)		
40–49 (1976–1967)	65/207 (31.4%)	142/207 (68.6%)		
50–59 (1966–1957)	53/204 (26.0%)	151/204 (74.0%)		
60+ (1956+)	45/148 (30.4%)	103/148 (69.6%)		
Sexual identity				
Gay (homosexual)	313/925 (33.8%)	612/925 (66.2%)	REF	REF
Bi (bisexual)	47/184 (25.5%)	137/184 (74.5%)	0.67 (0.47–0.96)	0.86 (0.48–1.53)
Other sexual orientations	45/138 (32.6%)	93/138 (67.4%)	0.95 (0.65–1.39)	1.70 (0.97–3.01)
Region				
Vancouver	346/1013 (34.2%)	667/1013 (65.8%)	REF	REF
Suburban Vancouver	37/156 (23.7%)	119/156 (76.3%)	1.31 (0.83–2.08)	0.83 (0.48–1.46)
Rest of BC	28/99 (28.3%)	71/99 (71.7%)	0.79 (0.45–1.40)	1.10 (0.46–2.66)
Ethnic/cultural identity				
White	286/872 (32.8%)	586/872 (67.2%)	REF	REF
Racialized minorities	116/372 (31.2%)	256/372 (68.8%)	0.93 (0.72–1.21)	0.81 (0.57–1.16)
Education				
< College	123/409 (30.1%)	286/409 (69.9%)	REF	REF
College+	288/859 (33.5%)	571/859 (66.5%)	1.17 (0.91–1.51)	1.08 (0.77–1.51)
Relationship status				
Single	206/655 (31.5%)	449/655 (68.5%)	REF	REF
Partnered with a man	157/443 (35.4%)	286/443 (64.6%)	1.12 (0.93–1.54)	1.04 (0.75–1.44)
Partnered with a woman	34/131 (26.0%)	97/131 (74.0%)	0.76 (0.50–1.17)	1.04 (0.48–2.26)
Survey recruitment location				
Community venues	90/304 (29.6%)	214/304 (70.4%)	REF	REF
HIM clinics	111/303 (36.6%)	192/303 (63.4%)	1.38 (0.98–1.93)	1.18 (0.76–1.81)
Online	210/661 (68.2%)	451/661 (68.2%)	1.11 (0.82–1.49)	1.01 (0.69–1.50)

Category counts may not add to 1272 due to missing data

^a^Question asked participants to select all that apply; thus, options were analyzed as binary variable

The following variables remained positively associated with GCO awareness in multivariable analysis, with aOR CI excluding 1: being out to one’s healthcare provider (aOR 1.92 (1.18–3.10)), frequent social media use (aOR 1.87 (1.10–3.17)), having visited an LGBT organization in the last 12 months (aOR 1.59 (1.14–2.21)), and usual testing at a public health STI clinic (aOR 1.57 (1.09–2.27)) or HIM clinic (aOR 1.64 (1.13–2.37)). Having tested at a hospital emergency room was negatively associated with GCO awareness (aOR 0.28 (0.10–0.84)).

Factors associated with intention to use GCO among those who were aware of the service but had not tested through GCO are shown in Table 3. Knowing other GCO users, using social media apps, not knowing where to test in person, not wanting to see a doctor, and all perceived benefits of the service, except having a physical exam, were associated with higher odds of intention to use, with uOR CI excluding 1. Knowing other GCO users (aOR 2.47 (1.02–5.97)) and the perceived benefit of GCO of saving time (aOR 2.62 (1.30–5.27)) remained associated with intention to use and statistically significant in multivariable analyses.

**Table 3:** Analyses with “Intention to use GCO” as the outcome restricted to those aware of the service and who have not tested through GCO (*n* = 367)

Predictor variables	*n/N* (%)^a^	uOR (95% CI)	aOR (95% CI)
Know other GCO users	36/58 (62.1%)	2.18 (1.22–3.89)	2.47 (1.02–5.97)
Satisfied with STBBI testing location			
Unsatisfied	28/49 (57.1%)	REF	REF
Satisfied	138/318 (43.4%)	0.58 (0.31–1.06)	1.33 (0.46–3.81)
Benefits: do not need to talk about my sex life	50/85 (58.8%)	2.00 (1.22–3.28)	1.67 (0.69–4.01)
Benefits: do not need to get a physical exam	29/54 (53.7%)	1.46 (0.82–2.61)	1.05 (0.35–3.15)
Benefits: easier to go to a lab than a clinic	72/114 (63.2%)	2.84 (1.79–4.49)	2.40 (1.11–5.21)
Benefits: getting test results online	89/158 (56.3%)	2.16 (1.41–3.30)	0.81 (0.39–1.70)
Benefits: testing without waiting for an appointment	101/182 (55.5%)	2.24 (1.47–3.42)	1.80 (0.82–3.94)
Benefits: testing when the clinic is full	62/115 (53.9%)	1.62 (1.04–2.54)	1.22 (0.53–2.77)
Benefits: do not need to see a doctor or nurse	63/111 (56.8%)	1.90 (1.21–2.99)	0.73 (0.32–1.69)
Benefits: saves time	79/130 (60.8%)	2.61 (1.68–4.06)	2.62 (1.30–5.27)
Drawbacks: not speaking with a doctor or nurse	65/163 (39.9%)	0.68 (0.45–1.04)	1.12 (0.57–2.20)
Drawbacks: getting test results online	5/11 (45.5%)	1.02 (0.31–3.40)	0.44 (0.06–2.98)
Drawbacks: need to print the lab form	27/67 (40.3%)	0.79 (0.46–1.35)	0.58 (0.24–1.37)
Drawbacks: not easy to get to a lab	12/33 (36.4%)	0.67 (0.32–1.41)	0.78 (0.25–2.39)
Drawbacks: not comfortable going to a lab	10/34 (29.4%)	0.48 (0.22–1.03)	0.57 (0.18–1.75)
Drawbacks: low trust in the service	5/19 (26.3%)	0.42 (0.15–1.19)	0.66 (0.13–3.25)
Drawbacks: worried about the privacy of my online information	24/63 (38.1%)	0.71 (0.41–1.24)	0.96 (0.37–2.46)
Drawbacks: need to have a promo code to sign up	20/34 (58.8%)	1.85 (0.90–3.79)	1.70 (0.51–5.65)
Testing barrier: did not know where to go	14/19 (73.7%)	3.69 (1.30–10.48)	1.94 (0.40–9.52)
Testing barrier: needed an appointment	27/51 (52.9%)	1.47 (0.81–2.66)	0.97 (0.38–2.51)
Testing barrier: the wait was too long	33/64 (51.6%)	1.39 (0.81–2.40)	0.91 (0.36–2.30)
Testing barrier: the clinic was too far away	12/20 (60.0%)	1.92 (0.76–4.81)	2.21 (0.44–11.08)
Testing barrier: the clinic wasn’t open when I could test	29/59 (49.2%)	1.23 (0.70–2.16)	0.76 (0.27–2.16)
Testing barrier: did not want to see a doctor or nurse	11/13 (84.6%)	7.21 (1.57–33.02)	8.28 (0.73–93.42)
Testing barrier: I do not like needles	6/9 (66.7%)	2.52 (0.62–10.24)	1.86 (0.17–20.57)
Testing barrier: could not get anonymous testing	9/15 (60.0%)	1.90 (0.66–5.45)	1.61 (0.27–9.69)
Out to healthcare provider			
Not out to healthcare provider	37/72 (51.4%)	REF	REF
Out to healthcare provider	129/295 (43.7%)	0.74 (0.44–1.23)	0.96 (0.34–2.72)
No. of sex partners in past year	Median: 6 (SD: 27.1)	1.00 (0.99–1.01)	1.00 (0.98–1.01)
Plays PokemonGo (a popular app in 2016)	44/94 (46.8%)	1.08 (0.67–1.72)	0.90 (0.40–2.03)
Cell use score		1.21 (0.97–1.50)	1.19 (0.79–1.79)
Hook-up app use	89/196 (45.4%)	1.02 (0.67–1.53)	1.15 (0.54–2.42)
Social media app score		1.18 (1.09–1.27)	1.01 (0.79–1.29)
Social media frequency			
Infrequent use	14/41 (34.1%)	REF	REF
Frequent use	152/326 (46.6%)	1.69 (0.85–3.33)	1.74 (0.58–2.42)
Age (no. of years)	Median: 35 (SD: 13.8)	1.00 (0.98–1.01)	1.01 (0.97–1.04)
Sexual identity			
Gay	127/284 (44.7%)	REF	REF
Bisexual	20/40 (50.0%)	1.24 (0.64–2.40)	0.72 (0.20–2.61)
Other sexual orientations	16/37 (43.2%)	0.94 (0.47–1.88)	0.45 (0.13–1.60)
Ethnicity/cultural identity			
White	113/253 (44.7%)	REF	REF
Racialized minorities	48/105 (45.7%)	1.04 (0.66–1.65)	1.02 (0.46–2.25)
Education: college+			
< College	51/108 (47.2%)	REF	REF
College+	115/259 (44.4%)	0.89 (0.57–1.40)	1.02 (0.52–1.99)
Relationship status			
Single	184/354 (52.0%)	REF	REF
Partnered with a man	142/354 (40.1%)	1.08 (0.49–2.40)	0.62 (0.31–1.23)
Partnered with a woman	28/354 (7.9%)	0.80 (0.35–1.80)	0.63 (0.13–3.08)
Survey recruitment location			
Community venues	43/86 (50.0%)	REF	REF
HIM clinics	45/105 (42.9%)	0.75 (0.42–1.33)	1.02 (0.43–2.42)
Online	78/176 (44.3%)	0.80 (0.48–1.34)	0.65 (0.28–1.50)

^a^*n/N* = number who intend to use/number who do and do not intend to use

## Discussion

Two years after the implementation of a novel online STBBI testing service in British Columbia, Canada, we assessed awareness of GCO and intention to use among those aware of the service among a large sample of GBMSM. Overall, 32.4% of our sample were aware of GCO. Among those aware who had not yet used the service, 45.2% of GBMSM reported intending to test through GCO in the future. Factors associated with awareness included gay identity, usually testing at an STBBI clinic, being out to a healthcare provider, attending GBMSM venues, and frequent social media use. These findings are largely consistent with the GCO promotional strategy which targeted gay men during the first year post-launch that advertised online and in clinics (Gilbert et al. 2019). Intention to use GCO was associated with knowing other GCO users, using social media, not knowing where else to test, and not wanting to see a doctor. This is one of the first studies to examine intention to use among persons aware of a recently launched online STBBI testing service in the context of other testing options, rather than intention to use a hypothetical model (Gilbert et al. 2013; Hottes et al. 2012) or currently available online testing methods more broadly (Koekenbier et al. 2008; Platteau et al. 2015). This is also the first study to examine intention to use an existing online service within Canada. Below, we offer an interpretation of the results, specifically attending to how they will inform promotion of the GCO service in the future.

While our estimated uptake of GCO among GBMSM at the time of data collection was 3% of GBMSM in BC, our finding that 45.2% of survey respondents who were aware of GCO intended to use the service in the future suggests there are large opportunities for future promotion and expansion of the service. Our results specifically highlight opportunities to equitably target future promotion of GCO to those who were less likely to be aware of the service. This includes bisexual men, those who are not out to healthcare providers, those without a history of STBBI testing, and those who are less connected to GBMSM community venues. These groups are less likely to be reached through promotions targeting gay men, and thus may require using novel and creative outlets, such as support groups for bisexual individuals and network-based promotion and recruitment strategies (Hartman 2011). Reaching GBMSM who are less connected to the gay community may also mean using more general promotional strategies, such as Google Ad campaigns. Reaching these individuals will require creative solutions; however, it is clearly feasible to reach them, given that they participated in the survey. Future studies may include qualitative research with these specific groups to understand their opinion of online testing services like GCO. One result of such an approach may be more equitable provision of online sexual health services to those who otherwise miss out on the opportunity for prompt diagnoses and treatment of STBBIs.

Our results additionally suggest promotional messages that are likely to resonate with those who are aware of and intend to use GCO in the future. These messages may be most effective if they emphasize the convenience and efficiency of the service (e.g., saving time), and the ability to get tested without seeing a doctor or a nurse. However, given that our sample of survey participants who were aware of GCO underrepresents bisexual men and those disconnected from gay community venues and STBBI testing, additional work will be needed to understand which perceived benefits are most important to these underrepresented groups in particular.

This study is limited by its cross-sectional design. We cannot assess temporality and infer the direction of causation between variables examined; for example, we cannot know if participants were aware of GCO before attending the venues where GCO was advertised. We relied upon a non-probability sample of GBMSM for this study; thus, participants may not be representative of the larger GBMSM population. However, 31.8% (14/44) of survey respondents who used GCO were under the age of 30 compared with 39.5% (185/468) of all GBMSM users from September 2014 to December 2016 (data not shown). We attempted to mitigate this limitation by recruiting our sample from different sites, including community venues, GBMSM-specific STBBI testing clinics, and websites and apps used by GBMSM. A final limitation is that all information collected for this study was self-reported. Both recall and social desirability bias may influence the responses participants gave. We tried to mitigate for this by enabling participants to fill out the survey on their own when at community venues, having it provided by clinic receptionists instead of study staff in HIM clinics, and making the survey available online. With regard to recall bias, we limited the recall period of questions to within the past year.

## Conclusion

Future research should include more studies on the impact online STBBI testing is having on overall STBBI prevalence and incidence within GBMSM, other target populations, or among all users. Given our findings that bisexual men, those not out to healthcare providers, and those who do not test at STBBI clinics were less likely to be aware of GCO, we further recommend additional research into how best to adapt interventions (or promotional efforts) to reach these subpopulations. Studies are also needed to assess awareness and reach of GCO and other online STBBI interventions in populations other than GBMSM. Finally, we recognize that there is a paucity of research on the health system impacts of online testing services that are also worthy of attention, including how these are integrated with and affect face-to-face testing services, and their cost-effectiveness.
